# Supplementary material for: Tauroursodeoxycholic Acid Inhibits Clostridioides difficile Toxin-Induced Apoptosis
Source: Infect Immun. 2022 Jul 7;90(8):e00153-22. doi: 10.1128/iai.00153-22 (PMC9387233; doi:10.1128/iai.00153-22)
Supplement: Supplemental file 1 — Fig. S1 to S3 and Tables S1 and S2. Download iai.00153-22-s0001.pdf, PDF file, 1.1 MB [file iai.00153-22-s0001.pdf]

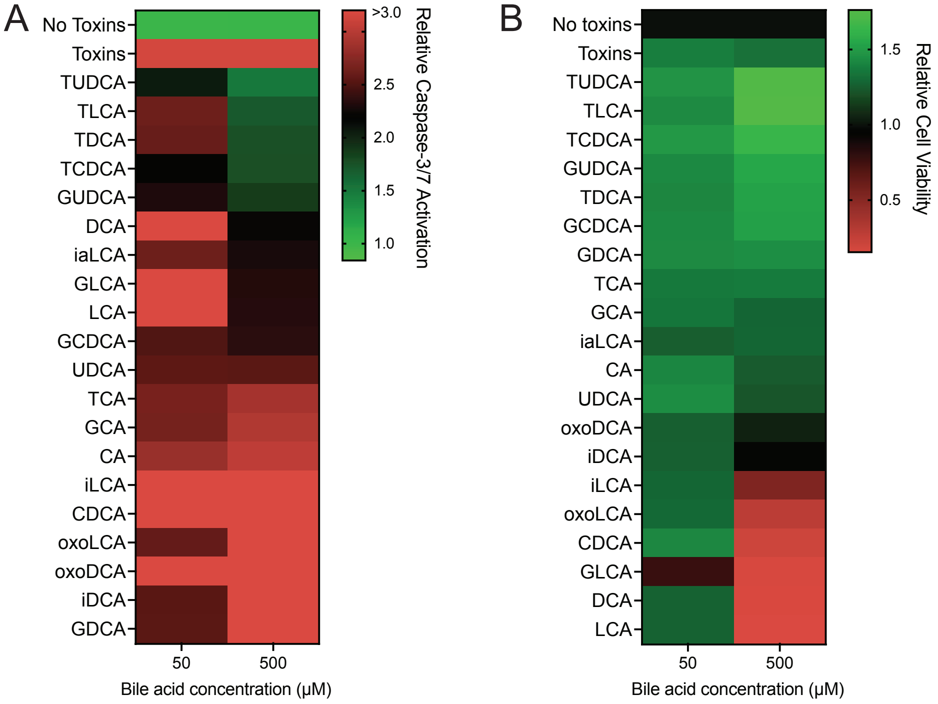

Supplemental Figure 1. (A) Relative caspase-3/7 activation and (B) relative cell viability in intoxicated Caco-2 cells 24 hr after treatment with the listed bile acids. Cells were pre-treated with toxins for 8 hr before the addition of bile acids. Data are relative to untreated cells. Bile acids are ordered based on (A) lowest caspase activation and (B) highest cell viability. The colors on the heatmap represent low (green) to high (red) relative caspase activation or low (red) to high (green) relative cell viability.

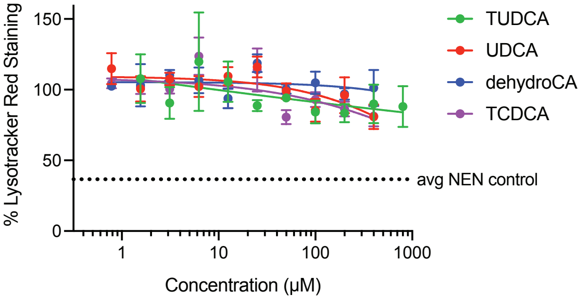

Supplemental Figure 2. TUDCA and UDCA do not affect lysosomal pH of IMR-90 cells, with EC50s >400μM. Bars represent SEM of three separate experiments. The dashed line represents percent of lysosomal staining for control compound Niclosamide (ethanolamine salt form; 4μM).

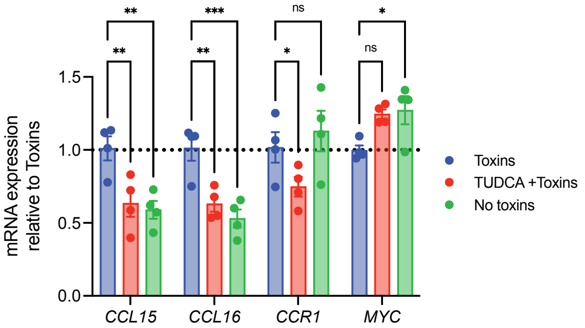

Supplemental Figure 3. Validation of differentially expressed Caco-2 transcripts by qRT-PCR. Expression was quantified from cDNA generated from RNA isolated from Caco-2 cells. Each point represents a biological replicate. All data are presented as the mean and error bars indicate the sd. * p <0.05, ** p < 0.005, *** p <0.0005; Studen't t test; n=4.

Supplemental Table 1. Bile acids used in this study.				
Name	**Abbreviation**	**Type**	**Vendor**	**Catalog Number**
Cholic Acid Sodium Salt	CA	Unconjugated primary	MP	M4412
Taurocholic acid	TCA	Taurine conjugated primary	Sigma	T4009
Glycocholic acid	GCA	Glycine conjugated primary	Sigma	360512
Sodium Chenodeoxycholate	CDCA	Unconjugated primary	Sigma	C8261
Sodium Taurochenodeoxycholate	TCDCA	Taurine conjugated primary	Sigma	T6260
Sodium glycochenodeoxycholate	GCDCA	Glycine conjugated primary	Sigma	G0759
Deoxycholic acid	DCA	Unconjugated secondary	Steraloids	C1070

Sodium Taurodeoxycholate	TDCA	Taurine conjugated secondary	Sigma	T0875
Glycodeoxycholic acid	GDCA	Glycine conjugated secondary	Sigma	G9910
Lithocholic acid	LCA	Unconjugated secondary	Steraloids	C1420
Taurolithocholic acid	TLCA	Taurine conjugated secondary	Steraloids	C1470
Sodium glycolithocholic acid	GLCA	Glycine conjugated secondary	Steraloids	C1437
Ursodeoxycholic acid	UDCA	Unconjugated secondary	Steraloids	C1020
Sodium Tauroursodeoxycholate	TUDCA	Taurine conjugated secondary	Sigma	T0266
Glycoursodeoxycholic Acid	GUDCA	Glycine conjugated secondary	Sigma	6863

isodeoxycholic acid	isoDCA	Unconjugated secondary	Steraloids	C1170
3-oxodeoxycholic acid	3-oxo-DCA	Unconjugated secondary	Steraloids	C1725
isoallolithocholic acid	isoalloLCA	Unconjugated secondary	Steraloids	C0700
3-oxolithocholic acid	3-oxo-LCA	Unconjugated secondary	Steraloids	C1750

Supplemental Table 2. Strains, plasmids and primers used in this study.		
Strain	**Genotype and Description**	**Reference**
R20291	Wild-type *C. difficile* strain from UK outbreak (ribotype 027)	(20)
R20291 Δ*tcdR*	Derivative of R20291 that lacks the *tcdR* gene	(35)
R20291 pDSW1728-P*tcdA*::*mCherryOpt*	R20291 carrying the pDSW1728-PtcdA:: *mCherryOpt* plasmid	this study
Plasmids		
pDSW1728	P*tet*::*mCherryOpt cat*	(63)
pDSW1728-P*tcdA*::*mCherry*	pDSW1728 derivative with P*tcdA*:: *mCherryOpt*	(63)

qRT-PCR Primers	
Target	**Primer 5′ to 3′**
Fw- *CCR1*	GGAGAGGGTCAGCTCCACATCTC
Rv- *CCR1*	CCCTCTCTATCCCAAGTGGC
Fw- *CCL15*	CAAGCCAGGTGTCATATTCCTC
Rv- *CCL15*	AACTCACAGGAGGTGTTGGA
Fw- *CCL16*	CAACCCCAGCTCCTCAACTCCCAG
Rv- *CCL16*	CATTGTTCTGCTTCTCTCAATGTG
Fw- *MYC*	GCTGCTTAGACGCTGGATT
Rv- *MYC*	CACCGAGTCGTAGTCGAGGT
